# Milling and fermentation influence mineral and phytic acid retention in zinc-biofortified wheat

**DOI:** 10.3389/fpls.2026.1954962

**Published:** 2026-10-07

**Authors:** Maria Itria Ibba, Velu Govindan

**Affiliations:** International Maize and Wheat Improvement Center (CIMMYT), Texcoco, Mexico

**Keywords:** fermentation, iron, mineral bioavailability, mineral retention, phytic acid, wheat biofortification, zinc

## Abstract

Wheat biofortification is an effective strategy to reduce micronutrient deficiencies. High retention and bioavailability of these micronutrients during processing is crucial. This study aimed to evaluate the effects of flour extraction rate and food processing on Zn, Fe, and phytic acid (PA) concentrations and indicators of potential mineral bioavailability in 60 Zn-biofortified wheat lines milled at 100%, 85% and 70% extraction rates (ER) and transformed into pan-bread and chapati. Reducing flour extraction rate from 100% to 70% decreased Zn, Fe, and phytic acid concentrations by approximately 70% (49.14 to 14.69 mg/kg), 49% (34.24 to 17.34 mg/kg), and 79% (0.97 to 0.20 g/100 g), respectively. Chapati and pan-bread production did not negatively impact Zn and Fe concentrations; however, PA content declined during processing, with greater reductions observed during pan-bread production due to PA degradation during fermentation. Pan-bread also showed lower phytate molar ratios, indicating improved potential mineral availability. The potential nutritional contribution of these products was evaluated in the context of Pakistan, where wheat is a major dietary staple and average wheat consumption is approximately 280 g/day. Based on this consumption level and theoretical Zn absorption estimates derived from the PA molar ratio, pan-bread produced from 100% ER flour was estimated to provide up to 149% of the physiological Zn requirement for women, while chapati produced from 85% ER flour was estimated to provide 95%.These results indicate that maintaining relatively high flour extraction rates can preserve Zn and Fe, while fermentation can reduce phytic acid and improve indicators of potential mineral availability, highlighting the importance of processing practices for maximizing the nutritional benefits of Zn-biofortified wheat in wheat-dependent populations such as Pakistan.

## 1 Introduction

Globally, around 372 million preschool-aged children and 1.2 billion non-pregnant women of reproductive age, suffer of micronutrient deficiency, also known as “hidden hunger” (Stevens et al., 2022). Among the different causes of hidden hunger, iron (Fe) and zinc (Zn) deficiencies are some of the most critical, because of their widespread presence across the globe and because of the physiological role these minerals play in the human body. Recent estimates indicate that micronutrient deficiencies remain highly prevalent worldwide, with Fe and Zn among the nutrients most commonly lacking in vulnerable populations (Stevens et al., 2022). Anaemia, which can result from iron deficiency among other causes, also remains a major global public health concern, affecting approximately 605 million women aged 15–49 years in 2023. Zinc deficiency, on the other hand, continues to be an important public health concern, particularly in low- and middle-income countries, where inadequate dietary Zn intake can contribute to impaired growth and development. Click or tap here to enter text.

Biofortification of edible crops intended as the improvement of key micronutrients either through classical breeding or agronomic methods, has been proven to be an appealing solution to increase micronutrient intake (Govindan et al., 2022; Li et al., 2024). Wheat biofortification specifically has focused on improving the grain Zn and Fe concentrations. HarvestPlus breeding targets define the additional grain micronutrient concentration required above an established baseline concentration to provide a nutritionally meaningful increase in micronutrient intake under typical consumption conditions. For wheat, a global baseline grain Zn concentration of approximately 25 mg/kg has been established, while the current HarvestPlus breeding target is an additional 12 mg/kg Zn above this baseline, corresponding to a target grain Zn concentration of approximately 37 mg/kg (Virk et al., 2021). According to the BSI PAS 233 specification (British Standards Institution (BSI), 2021), zinc-enriched wheat is further classified based on the increase in grain Zn concentration above this 25 mg/kg baseline as Class I (≥12 mg/kg above baseline; ≥37 mg/kg total Zn), Class II (9 to <12 mg/kg above baseline; ≥34 to <37 mg/kg total Zn), and Class III (6 to <9 mg/kg above baseline; ≥31 to <34 mg/kg total Zn). Breeding efforts by CIMMYT, HarvestPlus, and national partners have resulted in the release and adoption of 22 high-Zn wheat varieties in target countries, including India, Pakistan, and Mexico (https://www.harvestplus.org/crop/zinc-wheat/ accessed on the 30^th^ of July 2026).

Increasing the micronutrient content, however, might not be effective if common processing practices reduce the wheat’s mineral content and affect mineral bioavailability. For this reason, it is paramount to understand how wheat processing affects the concentration of Fe, Zn, and phytic acid (PA), the major component reducing mineral bioavailability (Rousseau et al., 2020).

Before consumption, wheat is milled into flour at variable extraction rates, which represent the percentage of flour obtained from the grain. The higher the extraction rate, the greater the amount of bran present in the flour (Bass, 1988); for example, whole wheat flour has a 100% extraction rate, while typical refined flour is around 70% and consists mostly of endosperm. Because minerals and bioactive components are concentrated in the bran, higher extraction flours offer better nutritional profiles. Conversely, refining wheat can reduce crucial minerals like Fe and Zn by up to 86% (Oghbaei and Prakash, 2016). However, the majority of PA is also concentrated in the bran, which can be reduced by lowering the flour extraction rate (Rousseau et al., 2020).

Bread is undoubtedly the major food product obtained from wheat flour, and a myriad of bread types are produced across the world. In general, bread can be divided into high-volume and flat bread and into fermented or unfermented bread. During the processing of flour into bread, the mineral content could either diminish because of leaching or increase because of the addition of other ingredients or transfer from machinery or packing material (Rousseau et al., 2020). Mineral bioavailability can also change during flour processing, especially when producing fermented bread. During fermentation, a drastic reduction in PA content (from 44% to up to > 90% reduction) has been reported in both sourdough and yeasted bread, primarily due to the pH-induced activity of endogenous phytase (Frontela et al., 2011; Buddrick et al., 2014).

Previous studies have demonstrated that milling and bread-making can substantially modify mineral and phytic acid concentrations in conventional and biofortified wheat (Ciccolini et al., 2017; Jiang et al., 2023; Wang et al., 2023). However, fewer studies have evaluated these effects across a large set of genetically Zn-biofortified breeding materials and simultaneously compared different flour extraction rates and contrasting fermented and unfermented wheat products. Thus, information remains limited on the combined effects of flour extraction rate and subsequent processing on mineral retention and phytic acid reduction in genetically Zn-biofortified breeding materials. We hypothesized that decreasing flour extraction rate would reduce both mineral and phytic acid concentrations due to the progressive removal of the outer grain layers, whereas fermentation would preferentially decrease phytic acid with comparatively little effect on Zn and Fe concentrations. Consequently, fermented bread will be expected to exhibit lower phytate:Fe or Zn molar ratios than unfermented chapati. For this reason, in the present study, a set of 60 Zn-biofortified wheat lines was used to investigate the retention of Fe, Zn, and phytic acid content in both fermented high-volume bread (pan-bread) and unfermented flat bread (chapati) obtained with three different extraction rate flours (100%, 85%, and 70%). The results of this study are key to better understanding the impact of wheat biofortification on the intake of such micronutrients and identifying possible new breeding targets or processing methods to maximize Fe and Zn retention and potential mineral bioavailability.

## 2 Materials and methods

### 2.1 Plant material

Sixty hard spring bread wheat lines derived from the CIMMYT bread wheat breeding program were used. All these lines were grown under full irrigation at the Campo Experimental Norman Ernest Borlaug (CENEB) station in Ciudad Obregon, Sonora, northwestern Mexico, during the cropping cycle 2018–2019. The lines were grown on raised beds with about 500 mm of available water and optimal sowing date during late November–early December. Sufficient fertilizers and water were supplied throughout the cycle to avoid potential nutrient and drought stresses. Fungicide and pesticides were applied to protect the crop from pest and diseases following the standard procedures at the station. The lines were selected based on a combination of desirable agronomic traits and high grain Zn concentration. All materials were experimental breeding lines, and no commercial checks were included. An overview of the experimental design, including flour extraction rates, processing pathways, sample types, and analytical determinations, is presented in **Figure 1**. Only one field plot per line was used for the analyses. The 60 wheat lines/field plots were considered independent biological/experimental units (n = 60), and the same 60 units were evaluated across the three flour extraction rates and corresponding processing pathways.

**Figure 1:**
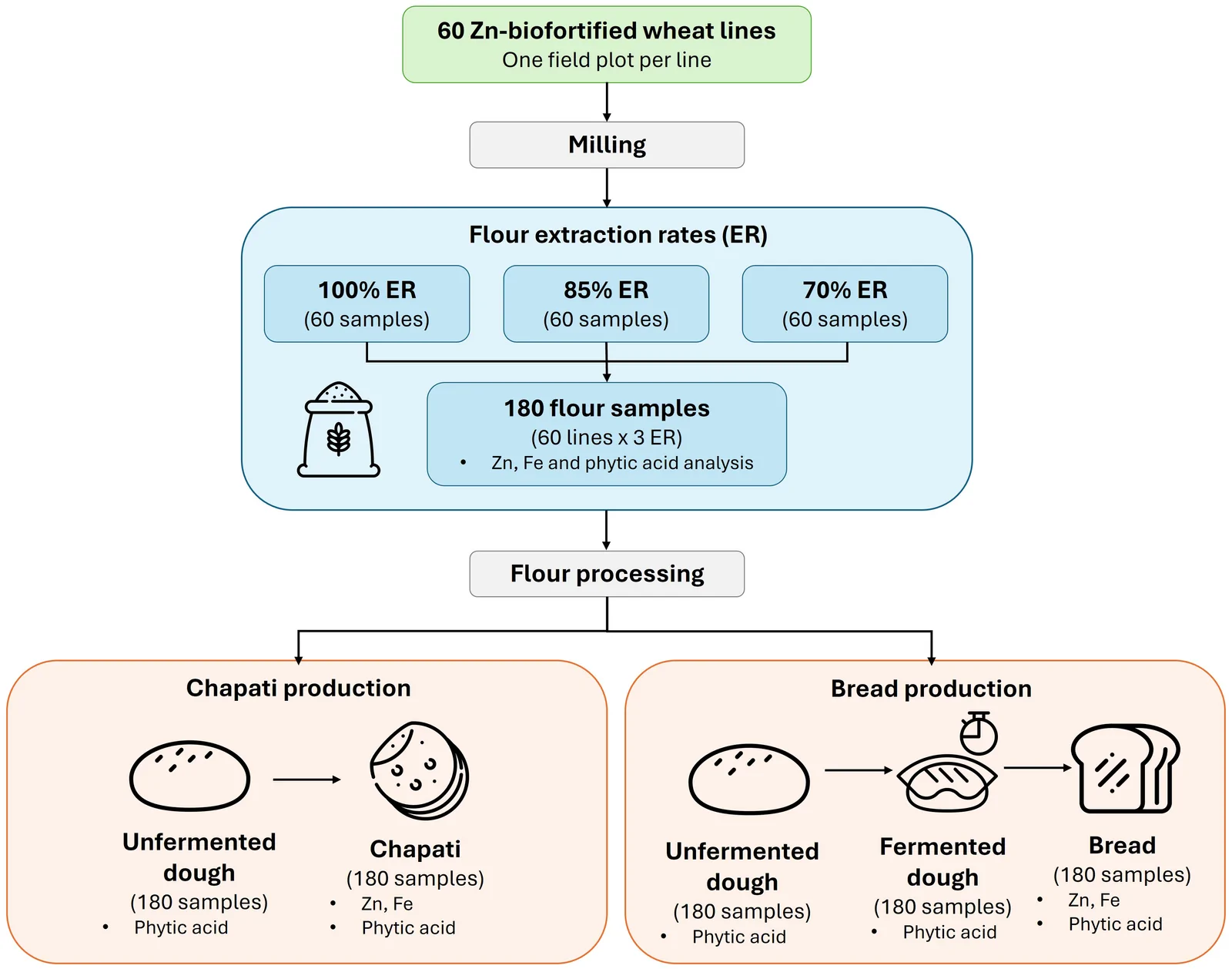
Overview of the experimental design and sample processing scheme. Sixty Zn-biofortified wheat lines, each represented by one field plot, were milled at three flour extraction rates (ER): 100%, 85%, and 70%, generating 180 flour samples (60 lines × 3 ER). Flour samples were analyzed for Zn, Fe, and phytic acid and subsequently processed into chapati and pan bread. Phytic acid was evaluated in unfermented chapati dough and in unfermented and fermented bread dough, while Zn, Fe, and phytic acid were determined in the final chapati and bread samples. All analytical determinations were performed in duplicate as technical replicates.

### 2.2 Grain physical and chemical analysis

Test weight (TW) was analyzed according to the AACC method 55-10.01 (AACC, 2011). Grain hardness was analyzed using the Single Kernel Characterization System (SKCS) instrument (SKCS Model 4100; Perten Instruments) following the AACC method 55-31.01 (AACC Approved Methods of Analysis, 2011). The SKCS was also used to determine the mean single-kernel weight, from which the thousand-kernel weight was calculated. Grain moisture (%) and grain protein (GPRO, 12.5% moisture basis) content were measured using near-infrared spectroscopy (DA 7200 NIR; Perten Instruments), with a calibration validated using the air-oven method for moisture and the Dumas combustion method (FP828 Leco^®^ Instruments) for protein and following the AACC official protocols 39-25.01, 44-15.02, and 46-30.01 (AACC, 2011).

### 2.3 Flour milling, protein and ash content analysis

Grain samples were tempered with water according to the official AACC method 26-95.01 (AACC, 2011) and milled into flour using a Bühler experimental mill (MLU-202, Bühler Holding AG, Switzerland) according to the AACC method 26-21.02 (AACC, 2011). Different flour extraction rates (ER) (70% and 85%) were obtained by changing the break roll spacing. Specifically, for the 70% ER flour, the first break roll was set to an approximate gap of 0.381 mm, while the second and third break rolls were set to approximately 0.102 mm. For the 85% ER flour, the first break roll was set to an approximate gap of 0.102 mm, while the second and third break rolls were set as closely as possible without allowing contact between the rolls. Following milling, the bran fraction was sieved for 2 min through an 8XX sieve (0.183 mm), and the remaining coarse fraction was passed through the reduction head of a Brabender Senior mill, sieved again for 2 min through an 8XX sieve, and recombined and thoroughly mixed with the flour. Whole wheat flour (100% ER) was obtained by milling non-tempered grain directly in the Bühler experimental mill set up for a 70% extraction rate and collecting all the obtained product. All flour samples were analyzed for ash content according to the AACC official method 08-12.01 (AACC, 2011). Moisture (%) and protein content (FPRO, 14% moisture basis) of the flour samples obtained with 70% and 85% extraction rates were analyzed using near-infrared spectroscopy (DA 7200 NIR; Perten Instruments), with a calibration validated using the air-oven method for moisture and the Dumas combustion method (FP828 Leco^®^ Instruments) for protein, and following the AACC official protocols 39-11.01, 44-15.02 46-30.01 (AACC, 2011).

### 2.4 Bread preparation

Bread was prepared following the official AACC method 10-10.03 (AACC, 2011) and using the following ingredients: 100 g of either 70%, 85% or 100% ER flour (14% moisture), 1 g of NaCl, 5 g of sugar and 3 g of fresh yeast. The water added to the different bread formulations was based on each flour water absorption capacity and was determined according to Guzmán et al. (2015). Dough samples at 0 min. fermentation and at 180 min. fermentation, and bread samples representative of all bread parts (crumb and crust) were lyophilized and ground using a Retsch Mixer Mill MM 400 fitted with zirconium grinding jars and balls (Retsch GmbH & Co KG, Germany).

### 2.5 Chapati preparation

Chapati were prepared based on the protocols reported by Haridas Rao et al. (1986) and Survase et al. (2017) with few modifications. Specifically, 200 g of either 70%, 85% or 100% ER flour were mixed with a variable quantity of water determined according to Guzmán et al. (2015). The obtained dough was let to rest for 15 minutes, rolled to uniform thickness (2 mm), and cut into 15 cm diameters using a circular aluminum die. The chapati was then baked on a thermostatically controlled hot plate at the maximum temperature for a total of 150 s (45 s on the first side and 105 s on the second side). Dough samples at 0 min. fermentation and chapati samples were then lyophilized and ground using a Retsch Mixer Mill MM 400 fitted with zirconium grinding jars and balls (Retsch GmbH & Co KG, Germany).

### 2.6 Determination of mineral content

Zn and Fe concentration was analyzed in all the flour, bread and chapati samples obtained with the three different flour extraction rates. The analyses were conducted in duplicate as technical replicates using inductively-coupled plasma-optical emission spectroscopy (ICP-OES) (7500cx; Agilent Technologies, USA) after a closed-tube nitric acid/hydrogen peroxide digestion method (Wheal et al., 2011). Briefly, approximately 300 mg of each sample was weighed into digestion tubes and 5 mL of an acid mixture was added. Samples were pre-digested overnight at room temperature and subsequently subjected to controlled acid digestion. After digestion and cooling, the mineral residues were dissolved in 1% nitric acid and quantitatively transferred to plastic tubes, with a final volume of 20 mL. Fe and Zn concentrations were determined against an external calibration curve prepared using Fe and Zn standards. Analytical blanks and reference materials were included during the analysis for quality control. Aluminum was analyzed as an indicator of potential sample contamination (Palacios-Rojas, 2018). Mineral concentrations were expressed as mg/kg of sample. Duplicate analytical determinations were considered technical replicates and were averaged to obtain one Fe and Zn value per sample before statistical analysis.

### 2.7 Determination of phytic acid content

Phytic acid (PA) content was analyzed in all the flour, dough, chapati and bread samples obtained with the three different flour extraction rates. The analyses were conducted in duplicate as technical replicates using the Megazyme Phytic Acid Assay kit (Megazyme Ltd., Ireland) with some modifications as reported by Posadas-Romano and Ibba (2022). Briefly, PA was extracted from the samples with 0.66 M hydrochloric acid overnight at room temperature. After centrifugation, an aliquot of the supernatant was neutralized with sodium hydroxide and adjusted with sodium acetate buffer (pH 5.5). The neutralized extracts were subjected to sequential enzymatic dephosphorylation using phytase and alkaline phosphatase to release inorganic phosphate from phytic acid and lower inositol phosphates. Free and total phosphorus reactions were performed separately, and the difference between them was used to estimate the phosphorus originating from phytic acid. Released phosphorus was quantified using a modified molybdenum-blue colorimetric assay. Absorbance was measured at 655 nm, and phosphorus concentration was determined from a calibration curve prepared with phosphorus standards. PA concentration was then calculated from the measured phosphorus content using a conversion factor of 0.282, corresponding to the proportion of phosphorus in phytic acid, and expressed as g/100 g of sample. Duplicate analytical determinations were considered technical replicates and were averaged to obtain one Fe and Zn value per sample before statistical analysis.

### 2.8 Calculation of the phytic acid:minerals molar ratios

Molar ratios between PA and Zn or Fe content in flour, chapati and bread, were calculated to estimate the bioavailability of such minerals (Norhaizan and Nor Faizadatul Ain, 2009). To calculate the molar ratios of phytic acid:iron (PA: Fe) and phytic acid:zinc (PA: Zn), the contents of PA, Fe and Zn, were converted into moles by dividing their concentrations by their respective molar mass and atomic weight (660.04, 55.85 and 65.4 g/mol, respectively). Values of PA: Fe > 1 and PA: Zn > 15 were used as indicators of the low bioavailability (<15% absorption) of such minerals (Gibson et al., 2010; World Health Organization (WHO), 1996).

### 2.9 Assessment of leavened and flat bread contribution to daily Zn intake and estimated Zn absorption

The potential contribution of pan bread and chapati made from biofortified wheat, milled at three different flour extraction rates, to the daily intake of Zn and the Zn Estimated Average Requirement (EAR), was calculated using the daily wheat consumption in Pakistan as a reference. Pakistan is one of the major target countries for Zn-biofortified wheat and according to 2022 data from FAOSTAT (Food and Agriculture Organization of the United Nations, 2022), the average daily wheat consumption is 280 g. An EAR for Zn of 2969 μg/day for women of childbearing age and 3544 μg/day for adult men was used as a reference following the recommendations of the European Food Safety Authority (European Food Safety Authority NDA Panel, 2014). Potential Zn absorption was estimated using the phytate molar ratio as a screening indicator. Based on the categories used in the referenced WHO framework (World Health Organization (WHO), 1996), samples with phytate molar ratios between 5 and 15 were assigned an assumed fractional Zn absorption of 30%, whereas samples with ratios >15 were assigned an assumed fractional absorption of 15%. Estimated absorbed Zn was calculated as: Zn concentration × quantity of product consumed × assumed fractional absorption. These calculations represent theoretical estimates rather than direct measurements of intestinal Zn absorption. PA (World Health Organization (WHO), 1996).

### 2.10 Statistical analyses

Descriptive statistics, including mean, standard deviation, minimum, and maximum values, were calculated for all measured traits. For ash, protein, Zn, Fe, and phytic acid measured in flour samples, a one-way ANOVA was performed to evaluate the effect of flour extraction rate (n = 60 samples per extraction rate). For Zn, Fe, phytic acid, phytate:Zn molar ratio, and phytate:Fe molar ratio, the effects of flour extraction rate, product type, and their interaction were evaluated by two-way analysis of variance (n = 60 samples per extraction rate × product combination). Analyses were performed using the General Linear Model procedure (PROC GLM) in SAS OnDemand for Academics (SAS/STAT^®^, SAS Institute Inc., NC, USA). When significant differences were detected, means were compared using Fisher’s least significant difference (LSD) test at P < 0.05. Pearson correlation coefficients were calculated using PROC CORR in SAS OnDemand for Academics to evaluate relationships among all measured traits across flour extraction rates and products.

## 3 Results

### 3.1 Physical and chemical characteristics of the wheat grain samples

All the 60 samples analyzed in the present study exhibited good grain quality, with average test weight and thousand kernel weight values of 80 kg/hL and 47.6 gr, respectively. Most samples had hard to semi-hard kernels with recorded SKCS HI values typically greater than 45. The only exception was one sample which had semi-soft kernels (SKCS HI = 38.9). Slight variations in protein content were recorded, with values ranging from 12.1% to 15.2% (**Supplementary Table 1**).

### 3.2 Effect of milling on flour chemical composition

Before analyzing the effect of the chapati or bread production on the micronutrient retention and its estimated bioavailability, the effect of the different milling extraction rates on the flour composition was analyzed. In all cases, the extraction rate significantly influenced the flour chemical composition (**Supplementary Table 2**) and both protein, ash, mineral and phytic acid content gradually declined with the reduction in the rate of flour extraction (**Table 1**). A 5% and 10% decrease in flour protein content was observed in flour samples obtained respectively with 85% ER and 70% ER, compared with the average protein content detected in whole wheat flours. A more severe decrease between flours could be observed for ash (indicative of the overall flour mineral content) and zinc content. In both cases in fact, flours obtained with 85% and 70% ER exhibited, respectively, an average 40% and 70% decline in ash and zinc content compared to the values reported for whole wheat flour. Flour iron concentration also declined with the decrease of the flour extraction rate, even if the differences observed between the 85% or 70% ER flours and the whole wheat flour samples were not as severe as those observed for ash and zinc content (**Table 1**). Phytic acid was the component to be more strongly affected by the flour milling methods with flours obtained with 85% ER and 70% ER, exhibiting respectively a 47% and 79% decrease in phytic acid content compared to whole wheat flours.

**Table 1:** Average and standard deviation values of different components detected in the flour, dough, chapati and bread samples obtained using three milling extraction rates.

Trait	Product	100% Extraction Rate	85% Extraction Rate	70% Extraction Rate
Protein Content (%, 14% mb)^a^	Flour	13.72 ± 0.76^A^	12.97 ± 0.75^B^	12.28 ± 0.63^C^
Ash Content (%)	Flour	1.39 ± 0.06^A^	0.81 ± 0.06^A^	0.42 ± 0.02^A^
Zinc (mg/Kg)	Flour	49.14 ± 4.49^C^	29.75 ± 2.48^E^	14.69 ± 1.59^H^
	Chapati	53.45 ± 3.87^A^	33.29 ± 2.56^D^	16.66 ± 1.84^G^
	Bread	52.16 ± 4.20^B^	32.26 ± 2.58^D^	17.79 ± 1.73^F^
Iron (mg/Kg)	Flour	34.24 ± 3.20^B^	30.93 ± 8.69^D^	17.34 ± 3.80^F^
	Chapati	35.02 ± 2.73^B^	30.12 ± 7.50^D^	20.06 ± 3.76^E^
	Bread	37.10 ± 2.99^A^	32.67 ± 6.18^C^	17.48 ± 3.42^F^
Phytic Acid (g/100g)	Flour	0.97 ± 0.08^A^	0.51 ± 0.05^G^	0.20 ± 0.02^L^
	UCD	0.83 ± 0.08^C^	0.41 ± 0.05^I^	0.14 ± 0.03^M,N^
	Chapati	0.85 ± 0.07^B^	0.46 ± 0.05^H^	0.15 ± 0.02^M^
	UBD	0.72 ± 0.09^D^	0.31 ± 0.04^J^	0.14 ± 0.01^M,N^
	FBD	0.64 ± 0.08^F^	0.23 ± 0.05^K^	0.14 ± 0.01^M,N^
	Bread	0.67 ± 0.08^E^	0.23 ± 0.04^K^	0.13 ± 0.01^N^
PA:Zn	Flour	19.73 ± 1.82^A^	17.17 ± 1.57^B^	13.65 ± 1.73^D^
	Chapati	15.85 ± 1.55^C^	13.94 ± 1.15^D^	9.29 ± 1.19^F^
	Bread	12.69 ± 1.22^E^	7.04 ± 0.97^H^	7.56 ± 0.79^G^
PA:Fe	Flour	24.21 ± 2.42^A^	14.27 ± 3.35^D^	10.22 ± 1.85^E^
	Chapati	20.72 ± 2.05^B^	13.94 ± 2.05^D^	6.74 ± 1.08^F^
	Bread	15.26 ± 2.10^C^	6.08 ± 1.12^F^	6.78 ± 1.09^F^

UCD, Unfermented chapati dough; UBD, unfermented bread dough; FBD, fermented bread dough; PA:Zn, Phytic acid:zinc molar ratio; PA:Fe, Phytic acid:iron molar ratio.

^a^ For the 100% extraction rate, the protein content was recorded on the grain and expressed at 12.5% moisture basis. Values represent the mean of 60 independent wheat lines (n = 60) for each extraction rate × product combination. Values followed by different letters are significantly (*p* < 0.05) different within each component.

### 3.3 Effect of chapati production on zinc, iron and phytic acid retention

Production of chapati did not appear to negatively influence mineral content. Across the different extraction rates, both the Zn and Fe content detected in the chapati were similar to or higher than the content of the same minerals detected in the flours used to produce this flatbread (**Supplementary Table 3**; **Figures 2A, B**). As expected, flour extraction rate was the major cause of variation among all the analyzed chapati, with samples obtained from 85% ER flour exhibiting on average a 38% and 14% reduction of Zn and Fe, respectively, compared to the chapati obtained with whole wheat flour. Similarly, samples obtained from 70% ER flour had on average 69% less Zn and 43% less Fe compared to the chapati obtained with whole wheat flour (**Table 1**).

**Figure 2:**
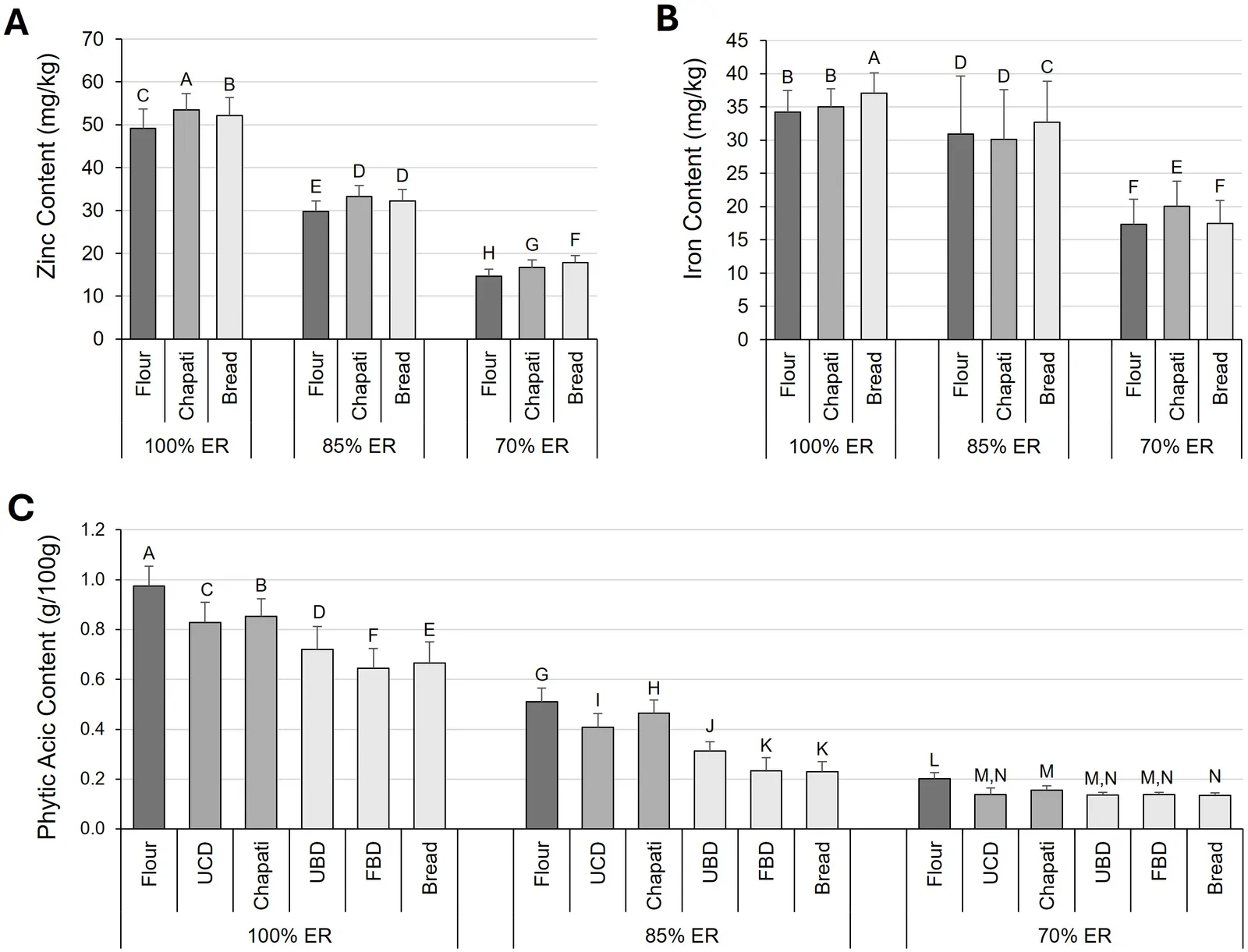
Changes in **(A)** zinc, **(B)** iron, and **(C)** phytic acid content across flour, unfermented chapati dough (UCD), chapati, unfermented bread dough (UBD), fermented bread dough (FBD), and bread made from flour with three different extraction rates (ER). Different letters above each bar indicate significant (*p* < 0.05) differences in zinc, iron, or phytic acid concentrations.

In contrast to the mineral content, slight but significant reductions in phytic acid were observed in both the chapati unfermented dough and the cooked chapati samples across all three different ERs (**Supplementary Table 3**; **Figure 2C**). These exhibited a reduction of ~10% to 25% in phytic acid compared to the respective flours used to obtain the samples (**Table 1**). Interestingly, the PA content detected in the cooked chapati was slightly higher compared to the concentrations observed in the unfermented chapati dough. Independently from the chapati production process, flour ER was the most significant factor influencing the overall chapati dough and cooked chapati PA content. For both products, a PA reduction of around 46% and 82% was detected when using flour obtained with the 85% and 70% ER, respectively (**Table 1**; **Figure 2C**).

### 3.4 Effect of leavened bread production on zinc, iron and phytic acid retention

Like chapati, leavened bread production did not appear to negatively influence Zn and Fe concentrations. On the contrary, the mineral content observed in the analyzed breads was on average 3 mg/kg greater than the Zn and Fe content detected in the flour used to make these products (**Table 1**; **Figures 2A, B**). In line with the variations in mineral concentrations observed between flours with different extraction rates, breads made from 100% ER flours exhibited the highest Zn and Fe concentrations (52.16 mg/kg and 37.10 mg/kg), followed by breads made with 85% ER flours (32.26 mg/kg and 32.67 mg/kg) and 70% ER flours (17.79 mg/kg and 17.20 mg/kg).

PA appeared to be significantly and negatively influenced by bread production (**Supplementary Table 3**). A gradual reduction in PA was observed starting from the unfermented bread doughs, which exhibited 26%, 39%, and 30% less PA compared with, respectively, the 100%, 85%, and 70% ER flours used to make them. A further 7% reduction in PA was observed in the fermented bread dough and bread samples made with the 100% ER flours, which had 0.64 g/100g and 0.67 g/100g of PA, respectively. Even greater PA reductions were observed among the fermented bread dough samples and bread samples made with the 85% ER flour, which exhibited a 15% decrease in PA compared to the respective unfermented bread dough samples. The only exceptions were the fermented bread dough samples and the bread samples made with the 70% ER flours, which mostly had the same PA concentrations as the respective unfermented bread dough samples (**Table 1**; **Figure 2C**). Similar to the results obtained for chapati, flour ER was the most significant factor influencing the PA concentration among all the analyzed bread and bread dough samples. The unfermented bread dough, fermented bread dough, and bread samples made with the 85% and 70% ER flours exhibited on average 62% and 80% less PA compared to the respective samples made with whole wheat flour, respectively (**Table 1**; **Figure 2C**).

### 3.5 Assessment of zinc and iron estimated bioavailability and zinc contributions to daily intake from pan bread and chapati

Similarly to the Zn, Fe, and PA content, the PA: Zn and PA: Fe molar ratio values were significantly influenced by both the flour extraction rate and flour processing. Interestingly, the effect of flour processing into chapati or bread had a much stronger impact on these ratios compared to the other analyzed traits. In the case of PA: Zn, it explained even more variation than the flour extraction rate (**Supplementary Table 3**).

Independently of the ER, by transforming the flour into chapati, an average PA: Zn and PA: Fe reduction of 23% and 17%, respectively, was observed. Even greater changes in the PA: Zn and PA: Fe ratios were identified for leavened bread, which showed a 36% to 59.0% decrease in the PA: Zn molar ratio and a 34% to 57.0% decrease in the PA: Fe molar ratio compared to the values obtained for the respective flour samples (**Supplementary Table 3**; **Figure 3**). Flour extraction rate affected the PA: Zn and PA: Fe molar ratios differently in chapati and bread. The chapati samples showed a steady decline in both PA: Zn and PA: Fe values with a reduction in the flour ER (**Figure 3**). For bread, however, the lowest PA: Zn and PA: Fe values were detected when using the 85% ER flour. Compared to the bread samples obtained with 100% ER, those made with 85% ER flour exhibited a reduction in the PA: Zn and PA: Fe ratios of around 45% and 60%, respectively. This reduction was greater than that observed for the bread samples made with 70% ER flour, which showed a 40% reduction for PA: Zn and a 56% reduction for PA: Fe.

**Figure 3:**
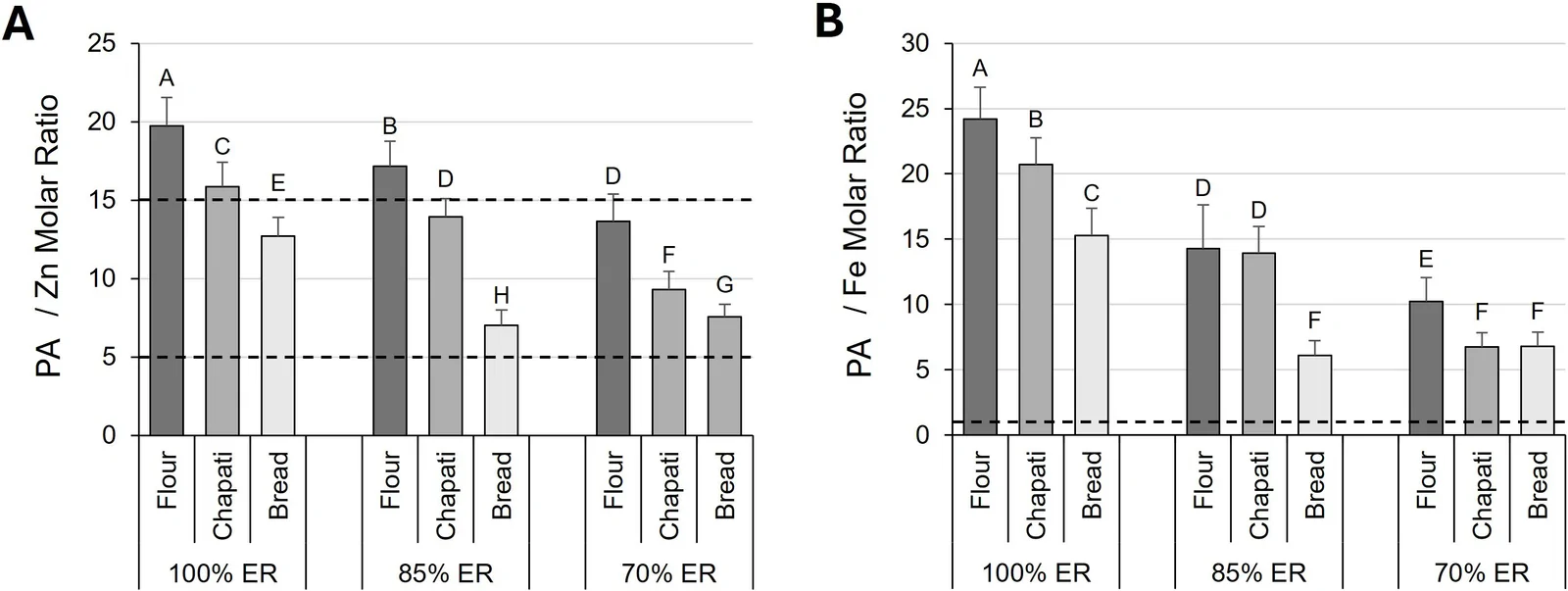
Changes in the **(A)** phytic acid: zinc, and **(B)** phytic acid: iron molar ratio across flour, chapati, and bread made from flour with three different extraction rates (ER). Different letters above each bar indicate significant (*p* < 0.05) differences in these ratios. Dotted lines highlight thresholds for **(A)** zinc low (> 15) and medium (> 5) bioavailability and **(B)** iron low bioavailability (> 1) (Gibson et al., 2010; World Health Organization (WHO), 1996).

Although phytate molar ratios were used to compare relative changes in potential mineral availability among products, Fe absorption was not directly quantified in this study. Therefore, conclusions regarding Fe are limited to changes in Fe concentration and the phytate molar ratio.

Once the PA: Zn molar ratio for pan bread and chapati were calculated and the estimated bioavailable percentage of Zn in these products using different extraction rates was understood, the contribution of these two products to the EAR of adult Pakistani women and men was determined (**Table 2**). Pan bread made from biofortified wheat contributed an estimated 51% to 149% of the Zn EAR for adult women and 42% to 125% of the Zn EAR for adult men, with a gradual increase observed as the flour extraction rate increased. A similar trend was observed for chapati made with either 70% or 85% ER flour (47% to 95% Zn EAR for women and 40% to 79% Zn EAR for men, respectively). The only exception was chapati made with 100% ER flour which was associated with high PA: Zn values (> 15) and low estimated Zn bioavailability (15%). This resulted in a lower overall estimated contribution to the daily Zn EAR for both women and men compared to the contribution of chapati made with 85% ER flour.

**Table 2:** Potential contribution of pan bread and chapati obtained from biofortified wheat milled at different extraction rates, to the zinc estimated average requirement (EAR) of adult women and men in Pakistan.

Product	Flour Extraction Rate (%)	Zn Content (ug/g)	Estimated bioavailability (%)^a^	Zn daily intake (ug)^b^	Estimated daily absorbed Zn (ug)	EAR % for adult women^c^	EAR % for adult men^c^
Bread	70	17.8	30	5017	1505	51	42
	85	32.3	30	9096	2729	92	77
	100	52.2	30	14709	4413	149	125
Chapati	70	16.7	30	4697	1409	47	40
	85	33.3	30	9388	2816	95	79
	100	53.4	15	15073	2261	76	64

^a^ Zinc bioavailability was based on the molar ratio between Zn and phytic acid, and the associated estimated % of bioavailability reported by World Health Organization (WHO), 1996.

^b^ The estimated daily wheat and wheat products intake used for this study was of 280 g for both men and women and was obtained using 2022 data from FAOSTAT.

^c^ EFSA estimated average daily Zn physiological requirement = 2969 ug for women and 3544 ug for men.

## 4 Discussion

Processing can significantly impact the nutritional quality of food, including the concentration, bioaccessibility, and bioavailability of key micronutrients like Fe and Zn. Zinc (and indirectly iron) biofortified wheat has been identified as a crucial measure to improve nutrition security for vulnerable populations in several low- and middle-income countries (Lowe et al., 2022). Understanding how wheat processing affects the bioavailability of these key micronutrients is essential to evaluate the effectiveness of wheat biofortification.

In this study, we analyzed 60 high-zinc spring bread wheat lines. The grains were milled at different extraction rates and processed into two common wheat-based products, pan bread and chapati, to evaluate the effects of milling and processing on micronutrient retention and their potential bioavailability.

As expected, neither the production of bread nor chapati significantly changed micronutrient content. Indeed, micronutrients are not destroyed by light, heat, or humidity (Rousseau et al., 2020). However, slight increases in the concentration of such micronutrients were observed in the final product, likely determined by contamination during processing and, in the case of bread, the utilization of yeast. These results align with previous observations in wheat and other biofortified crops (Cubadda et al., 2009; Heshe et al., 2016; Taleon et al., 2020; Gallego-Castillo et al., 2021). In contrast, the milling process significantly impacted micronutrient retention, with a gradual reduction of both Fe and Zn as flour refinement increased (up to 49% and 70% reduction, respectively, in flour obtained using 70% ER). This reduction is particularly relevant when considered in relation to current Zn biofortification target. The average Zn concentration decreased from 49.14 mg/kg in whole wheat flour, a value above the HarvestPlus target of approximately 37 mg/kg grain Zn, to 29.73 and 14.69 mg/kg in flour obtained at 85% and 70% ER, respectively. Although the HarvestPlus breeding target and BSI PAS 233 classification apply to grain rather than processed flour, these results highlight that achieving the target Zn concentration at the grain level does not necessarily ensure retention of similarly elevated Zn concentrations after milling at lower than 100% extraction rate. Most minerals in wheat and cereal grains are concentrated in the bran (Shewry and Hey, 2015), which is primarily removed during flour refinement. Previous studies on wheat (Cubadda et al., 2009; Frontela et al., 2011; Heshe et al., 2016), maize (Gallego-Castillo et al., 2021), and rice (Taleon et al., 2020) also showed that milling and refinement significantly reduce key minerals and other micronutrients, sometimes by more than 70%.

Increases in micronutrient content are not necessarily associated with their enhanced bioavailability due to compounds in food that interfere with their absorption in the small intestine. Phytic acid, a well-known mineral antinutrient, forms stable complexes with Fe and Zn, known as phytates, which are not easily digestible by humans due to the lack of endogenous phytase enzymes (Rousseau et al., 2020). Consequently, the bioavailability of these bound ions is impaired. Reducing phytic acid through physical elimination or enzymatic degradation is a good option to increase bioavailability (Gupta et al., 2015). Given that phytic acid is concentrated in the outer layers of the grain, lower flour extraction rates are associated with decreased phytic acid. In this study, we observed average reductions of 47% and 79% in PA content in flour with 85% and 70% extraction rates, respectively, similar to results previously reported in wheat and in other crops (Frontela et al., 2011; Gallego-Castillo et al., 2021; Taleon et al., 2020). However, reducing flour extraction rates also removes other key micronutrients including Fe and Zn and is not the best technique to solely increase bioavailability (Rousseau et al., 2020).

Fermentation has been widely reported to increase micronutrient bioavailability due to the activation of endogenous phytases or the synthesis of microbial phytases (Gupta et al., 2015; Rousseau et al., 2020). Phytases catalyze the hydrolysis of phytic acid, increasing soluble minerals like Fe and Zn. In this study, the average reduction of phytic acid in bread (leavened product) compared to the flour was much greater (~39%) than in chapatis (~12%), confirming that fermentation significantly increases micronutrient bioaccessibility. These results are comparable to previous studies using similar bread-making processes but lower than reductions reported by Frontela et al. (2011), who observed PA reductions of 72% and 84% in whole wheat and refined bread, respectively.

However, the reduction of PA in bread did not result in PA molar ratios below the threshold associated with improved Fe absorptionPA (Gibson et al., 2010). Since Fe absorption was not directly quantified in this study, these ratios should be interpreted only as indicators of potential Fe availability. For Zn, all bread samples had a PA: Zn molar ratio between 5 and 15, indicating moderate Zn absorption (World Health Organization (WHO), 1996). Bread made with 85% ER flour had slightly lower ratios, possibly due to better balance between micronutrient and phytic acid content and increased phytate degradation from endogenous phytase activity. Chapati made with either 85% or 70% ER flour also had PA: Zn molar ratios between 5 and 15 indicating that, even without the additional phytate degradation associated with the fermentation process, moderate absorption of Zn could be achieved when using biofortified wheat varieties.

Since 2016, Zn-biofortified wheat has been used to help address the inadequate zinc intake in Pakistan, where more than 60 million people are estimated to consume a Zn-deficient diet (HarvestPlus, 2022). Indeed, through the combined efforts of CIMMYT, HarvestPlus, and their public- and private-sector partners, the biofortified variety Akbar-19 has been adopted on more than 40% of the wheat-growing area in Pakistan (HarvestPlus, 2024). Pakistan is also one of the leading wheat-consuming countries in the world, with an average daily per-capita consumption of 280 g of wheat (Food and Agriculture Organization of the United Nations, 2022). Given the widespread adoption of biofortified wheat by Pakistani farmers, and the crucial role that wheat plays in the national diet, Zn-biofortified wheat represents a promising strategy for addressing zinc deficiency in this country. Using the average quantity of wheat and wheat products consumed in Pakistan in 2022 (Food and Agriculture Organization of the United Nations, 2022) as an example, consumption of leavened bread made from 100% ER flour was associated with a daily Zn intake greater than the daily Zn physiological requirement reported by the EFSA. Nevertheless, chapati made from 100% ER flour is the main wheat product consumed in this country (Prikhodko and Zrilyi, 2013; Khurshid et al., 2020). Based on the results obtained, it would still provide 74% and 64% of the daily Zn physiological requirement for women and men, respectively. Slight refinement of the flour (80% to 90% ER), along with targeted reduction of phytic acid in biofortified wheat, could further increase the contribution of chapati to the daily Zn physiological requirement in this country. In comparison, non-biofortified wheat (baseline Zn concentration = 25 ppm, HarvestPlus), when consumed as leavened or non-leavened flatbread made from 100% ER flour, could theoretically provide only half of the Zn EAR% for adult women and men provided by biofortified wheat. This comparison further validates the efficacy of biofortifying wheat in a large wheat-consuming country like Pakistan.

From a commercial perspective, these results suggest that relatively high flour extraction rates, even without producing wholemeal flour, could represent a practical strategy to improve mineral retention in wheat-based products. Higher extraction-rate flours could be readily obtained in industrial milling systems by adjusting the process to retain a greater proportion of the bran fractions. However, their functional and sensory characteristics and acceptance by processors and consumers, particularly in applications traditionally based on refined flour, would need to be evaluated. Fermentation represents another commercially relevant opportunity, as many wheat-based products already undergo fermentation. Further studies should determine whether existing commercial fermentation conditions and times effectively reduce PA and improve indicators of potential mineral availability, and whether these processes could be optimized while maintaining product quality. Together, these approaches could enhance the nutritional benefits of Zn-biofortified wheat through feasible adaptations of existing milling and processing practices.

Overall, this study confirms the significant effect of milling on mineral concentration and estimated bioavailability. Bread production does not change Fe and Zn content and can increase micronutrient bioavailability depending on the process used. However, simply increasing mineral content in wheat may not be sufficient to improve micronutrient bioavailability in vulnerable populations. Additional biofortification targets should include reducing antinutrient components like phytic acid and selecting lines with increased endogenous phytase activity for better phytic acid degradation during dough fermentation. To reduce phytic acid in biofortified wheat and increase bioavailability, a proof-of-concept study identified potential genomic regions associated with high PA and breeding lines with low PA: Zn ratio (Wen et al., 2022). Similarly, low PA lines had been previously identified in an EMS-mutagenized wheat population (Guttieri et al., 2004) and the low-PA trait has been successfully introgressed into advanced winter wheat lines (Venegas et al., 2018). Perhaps, PA haplotypes should be used in genomic selection models and marker assisted breeding to select breeding lines with low PA and high Zn. In combination with nutritional improvements of Zn-biofortified wheat through breeding, promoting the consumption of bread and other food products that undergo a fermentation process could further enhance the efficacy of biofortified crops. An important limitation of this study is that mineral bioavailability was inferred from phytate molar ratios rather than measured directly. These ratios provide useful screening indicators of the potential inhibitory effect of phytate but do not account for other food-matrix components, gastrointestinal digestion, mineral speciation, host physiology, or intestinal transport. Therefore, the values reported here should not be interpreted as direct measurements of Zn or Fe bioavailability. Although a few efficacy studies show the health benefits of Zn biofortified wheat (Sazawal et al., 2018; Gupta et al., 2022, 2025; Lowe et al., 2022), future studies combining standardized *in vitro* digestion and bioaccessibility methods and, where appropriate, human absorption studies will be needed to confirm the nutritional implications of the processing effects observed in this study.as well as ideal targets for phytic acid content and phytase activity to maximize the effectiveness of crop biofortification.

## Data Availability

The original contributions presented in the study are included in the article/**Supplementary Material**. Further inquiries can be directed to the corresponding author.
